# Endoscopic ultrasound-guided therapies versus retrograde transvenous obliteration for gastric varices: Multicenter propensity matched analysis

**DOI:** 10.1055/a-2549-1165

**Published:** 2025-04-04

**Authors:** Suprabhat Giri, Ranjan Kumar Patel, Radhika Chavan, Bhavik Bharat Shah, Jimmy Narayan, Taraprasad Tripathy, Sushant Babbar, Lalit Garg, Rozil Gandhi, Karan Manoj Anandpara, Swati Das, Manjit Kanungo, Girish Kumar Pati, Hemanta K Nayak, Manas Kumar Panigrahi, Preetam Nath, Saroj Kanta Sahu, Dibya Lochan Praharaj, Bipadabhanjan Mallick, Sarat Chandra Panigrahi, Sanjay Rajput, Jimil Shah, Anil Chandra Anand, Manoj Kumar Sahu

**Affiliations:** 1 90946 Department of Gastroenterology and Hepatology, Kalinga Institute of Medical Sciences, Bhubaneswar, India; 2 410775 Department of Radiodiagnosis, All India Institute of Medical Sciences Bhubaneswar, Bhubaneswar, India; 3 609456 Gastroenterology, Ansh Clinic, Ahmedabad, India; 4 706422 Department of Gastroenterology, Shree Narayana Hospital, Raipur, India; 5 Department of Gastroenterology, MediGenix Hospital, Raipur, India; 6 250350 Department of Gastroenterology, SOA IMS and SUM Hospital, Bhubaneswar, India; 7 29761 Department of Interventional Radiology, Dayanand Medical College and Hospital, Ludhiana, India; 8 29783 Department of Interventional Radiology, Santokba Durlabhji Memorial Hospital, Jaipur, India; 9 Department of Interventional Radiology, Sushrut Hospital, Ahmedabad, India; 10 Department of Interventional Radiology, Heart & Vascular Superspecialty Hospitals, India, India; 11 90946 Department of Radiology, Kalinga Institute of Medical Sciences, Bhubaneswar, India; 12 410775 Department of Gastroenterology, All India Institute of Medical Sciences Bhubaneswar, Bhubaneswar, India; 13 609456 Department of Gastroenterology, Ansh Clinic, Ahmedabad, India; 14 29751 Gastroenterology, PGIMER, Chandigarh, India

**Keywords:** Endoscopic ultrasonography, Intervention EUS, GI radiology

## Abstract

**Background and study aims:**

Retrograde transvenous obliteration (RTO) is an established technique for managing fundal varices. Endoscopic ultrasound (EUS)-guided glue injection with or without coil is an alternate approach. The present study compared outcomes of EUS-guided therapies with RTO for managing fundal varices.

**Patients and methods:**

We retrospectively analyzed data from patients with fundal varices undergoing EUS-guided intervention or RTO at 10 tertiary centers in India and compared after propensity score matching. The primary outcome was variceal bleeding within 1 year. Secondary outcomes included procedure-related adverse events (AEs), variceal obliteration, reintervention, and mortality.

**Results:**

A total of 167 patients (EUS 108, RTO 59) were included, with 59 in each group after propensity score matching. Incidence of variceal bleeding (15.3% vs. 13.6%, *P* = 0.793) within 1 year was comparable between the groups. Procedure-related AEs were higher in the RTO group (22% vs. 5.1%, *P* = 0.007), primarily new onset or worsening of ascites. Variceal obliteration at 4 weeks was similar between groups (83.1% vs. 91.5%, *P* = 0.167). Although reintervention within 1 year of the index procedure (30.5% vs. 22.0%, *P* = 0.296) was comparable, the EUS group required more frequent reintervention for GVs (28.8% vs. 5.1%, *P* = 0.001), and the RTO group required more frequent reintervention for EVs (16.9% vs. 1.7%, *P* = 0.008).

**Conclusions:**

EUS-guided therapy offers a safe and effective alternative to RTO for managing fundal varices. Although reintervention rate for GVs were higher than for EUS, incidence of AEs and reintervention for EVs was higher with RTO.

## Introduction

Prevalence of gastric varices (GVs) and bleeding from GVs is lower than esophageal varices. However, GV bleeding is more severe than esophageal variceal bleeding and is associated with a higher incidence of mortality 1 . Despite advances in managing acute GV bleeding, selecting an optimal intervention for primary and secondary prophylaxis remains contentious 1 2 . Direct endoscopic injection therapy of tissue adhesive is still a widely used treatment for GVs. Alternative approaches, such as balloon-occluded retrograde transvenous obliteration (BRTO) or transjugular intrahepatic portosystemic shunt (TIPS), are recommended for severe or refractory bleeding based on availability and expertise 3 .

The Baveno VII guidelines recommend endoscopic tissue adhesives (e.g. N-butyl-cyanoacrylate/thrombin) injection for acute bleeding from isolated GV type 1 (IGV1), IGV2 and GVs type 2 (GOV2), whereas in GOV2, IGV1, and ectopic varices, BRTO or TIPS could be considered as an alternative to endoscopic treatment in selected cases 4 5 . However, these guidelines do not explicitly discuss endoscopic ultrasound (EUS)-guided therapy. Randomized controlled trials (RCTs) have reported the benefit of EUS-guided therapies over conventional endoscopic cyanoacrylate injection in terms of higher obliteration rate with a lower number of sessions and rebleeding 6 7 .

A network meta-analysis reported a comparable rebleeding rate with BRTO and EUS-guided therapies 8 . Despite growing evidence supporting EUS-guided intervention, direct comparisons with retrograde transvenous obliteration (RTO) remain scarce. This study aimed to evaluate and compare efficacy and safety of EUS-guided therapies with RTO for managing GV.

## Patients and methods

### Study design and patient selection

This multicenter, retrospective, observational study was conducted at 10 tertiary care centers in India after ethics clearance (KIIT/KIMS/IEC/1904/2024). All patients aged 18 to 75 years with fundal varices (GOV2 or IGV1) as defined by the Sarin classification undergoing variceal obliteration for an emergency indication, primary, or secondary prophylaxis from September 2020 to September 2023 were included. Exclusion criteria were 1) patients with prior shunt surgery; 2) associated ectopic varices; and 3) duration of follow-up less than 1 year.

### Procedure details

#### EUS-guided intervention

**Fig. 1:**
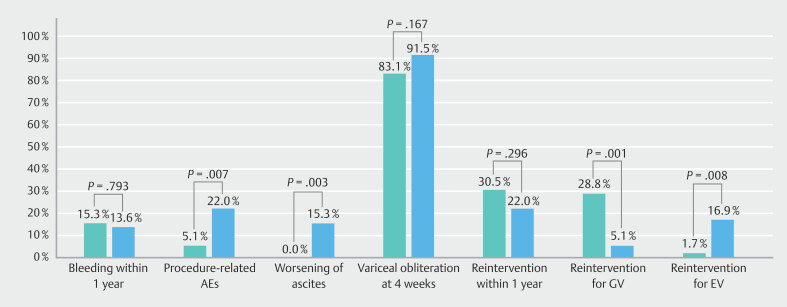
Clustered columns comparing primary and secondary outcomes between endoscopic ultrasound (EUS) and retrograde transvenous obliteration (RTO). Highlighted values indicate statistically significant differences.

All EUS-guided procedures were performed under deep sedation or general anesthesia by advanced endoscopists with more than 5 years of expertise in EUS-guided intervention. After visualization of the fundic varices or their feeder vessels on EUS, the puncture was taken from the transesophageal or transgastric route with a 19-G or 22-G fine-needle aspiration needle. Needle position was confirmed by either aspirating blood or injecting distilled water (1–2 mL). For glue-only injection (EUS-G), cyanoacrylate glue was injected into the varix under EUS guidance. For patients undergoing EUS-guided coil + glue (EUS-C+G), multiple coils (Nester Embolization Coils, Cook Medical) were deployed in the varices under EUS guidance after puncture ( Fig. 1 ). Size (0.035” for 19-G needle and 0.018” for 22-G needle), length (7–14 cm), diameter (10–18 mm), and number of coils were decided based on varix size and endoscopist discretion. After coil deployment, cyanoacrylate glue was injected into the varix 9 . Just mention obliteration of color flow on Doppler was documented.

#### Retrograde transvenous obliteration

All RTO procedures were performed by interventional radiologists with more than 3 years of expertise in gastrointestinal interventions. In all RTOs, variceal embolization was performed retrogradely via the efferent draining vein. In conventional BRTO, an appropriate-diameter occlusion balloon catheter was inserted in a retrograde fashion, either through the right femoral or jugular vein to the left renal vein. A retrograde venogram was obtained with the balloon inflated to occlude the shunt. A mixture of lipiodol, 3% sodium tetradecyl sulfate, and air (ratio 1:2:3) was injected to embolize the varices. The balloon catheter remained in situ for 4 to 24 hours, awaiting thrombus formation. Modified techniques included plug-assisted RTO (PARTO) and coil-assisted RTO (CARTO). In PARTO, a vascular plug was used instead of an indwelling occlusion balloon to shorten procedure time and avoid risk of balloon rupture. Similarly, in CARTO, multiple coils were used to occlude the efferent shunt. On a case-by-case basis, any small efferent vein, if noted during the procedure, was embolized using gelfoam slurry or coils to minimize non-target embolization. Depending on availability, intraprocedure cone beam computed tomography (CT) or a non-contrast CT on Day 1 was performed to evaluate deposition of sclerosant within the shunt and varices.

#### Follow-up

Patients were monitored in-hospital following the procedure, and adverse events (AEs) were recorded. Follow-up assessments were performed at 4 weeks or earlier if bleeding occurred using EUS and/or upper gastrointestinal endoscopy to confirm variceal obliteration. Subsequent evaluation occurred at 3-month intervals. We considered a window of 1 week on either side of the time point of the first assessment and 1 month for subsequent follow-up. Reintervention was performed as necessary for bleeding, incomplete obliteration, or new high-risk varices (esophageal or gastric).

### Definition of outcomes

The primary outcome was incidence of variceal bleeding (both esophageal and fundal) within 1 year after the procedure. Secondary outcomes included procedure-related AEs, variceal obliteration, reintervention, and mortality within 1 year. Procedure-related AEs were defined and graded as per the American Society of Gastrointestinal Endoscopy lexicon for endoscopy 10 and the Society of Interventional Radiology Specialty–Specific System 11 . Variceal obliteration was defined as absence of flow on Doppler in EUS view or hardening of varix on endoscopic assessment at 1-month follow-up. Reintervention was defined as repeat endoscopic or radiological intervention for bleeding, incomplete obliteration, or new high-risk varices.

### Statistical analysis

Descriptive statistics were expressed as mean with standard deviation for parametric data and median with interquartile range (IQR) for non-parametric continuous data. Categorical data were expressed as numbers (n, %). The Student’s *t* -test was used to compare continuous data. Pearson’s Chi-square test or Fisher’s exact test (as appropriate) was used to compare categorical data among the two groups. Propensity score matching was conducted using a logistic (logit) regression, in which 1:1 matching was done accounting for age, sex, cirrhosis status, and Child-Pugh score. We also conducted subgroup analysis based on procedure type and indication. Multivariate analysis with the logistic regression model was used to identify independent predictors of bleeding and reintervention. The main outcomes were reported as estimated effect sizes (odds ratio [OR]) along with precision (95% confidence intervals [CIs]). Statistical significance was set at *P* < 0.05. All statistical tests were performed using SPSS ver. 26.0 (IBM Corp., Armonk, New York, United States).

## Results

A total of 180 patients were treated for fundal varices, of whom 12 did not complete follow-up and one had associated ectopic varix. Finally, 167 patients were included in the analysis, of whom 108 patients (65.7%) underwent EUS-guided glue injection with or without coil (EUS-G 17 [10.2%] and EUS-C+G 91 [54.5%]) and 59 (35.3%) underwent RTO ([BRTO 14 [8.4%], PARTO 44 [26.3%], and CARTO 1 [0.6%]). Table 1 shows baseline characteristics of patients included in the study, with no significant difference between the groups except for proportion of patients with cirrhosis, which was significantly higher in the BRTO group (88.1% vs. 69.4%, *P* = 0.007). A 1:1 propensity matched comparison between EUS-guided and endoscopic management of GV yielded 59 pairs of patients in either group, which had comparable characteristics ( Table 1 ).

**Table:** **Table 1** Baseline patient characteristics in the overall and matched cohort.

Parameters	Overall			Matched		
	EUS (n = 108)	RTO (n = 59)	*P* value	EUS (n = 59)	RTO (n = 59)	*P* value
Median age (yr)	45 (18–75)	51 (18–75)	0.065	50 (18–75)	51 (18–75)	0.401
Male gender, n (%)	71 (65.7%)	46 (78.0%)	0.099	37 (62.7%)	46 (78.0%)	0.068
Cirrhosis, n (%)	75 (69.4%)	52 (88.1%)	**0.007**	44 (74.6%)	52 (88.1%)	0.059
Alcohol	35 (32.4%)	21 (35.6%)		18 (30.5%)	21 (35.6%)	
MASLD	18 (16.7%)	15 (25.4%)		14 (23.7%)	15 (25.4%)	
HBV	9 (8.3%)	3 (5.1%)		5 (8.5%)	3 (5.1%)	
HCV	4 (3.7%)	2 (3.4%)		3 (5.1%)	2 (3.4%)	
Others	9 (8.3%)	11 (18.6%)		4 (6.8%)	11 (18.6%)	
NCPH, n (%)	33 (30.5%)	7 (11.9%)	**0.007**	15 (25.4%)	7 (11.9%)	0.059
EHPVO	21 (19.4%)	2 (3.4%)		10 (16.9%)	2 (3.4%)	
NCPF	12 (11.1%)	5 (8.5%)		5 (8.5%)	5 (8.5%)	
Ascites, n (%)	37 (34.3%)	13 (22.0%)	0.099	20 (33.9%)	13 (22.0%)	0.151
Child-Pugh status						
A	59 (54.6%)	27 (45.8%)	0.273	29 (49.2%)	27 (45.8%)	0.712
B/C	49 (45.4%)	32 (54.2%)		30 (50.8%)	32 (54.2%)	
Indication, n (%)						
Primary	43 (39.8%)	16 (27.1%)	0.101	24 (40.7%)	16 (27.1%)	0.120
Secondary	65 (60.2%)	43 (72.9%)		35 (59.3%)	43 (72.9%)	
Varix type, n (%)						
GOV2	70 (64.8%)	41 (69.5%)	0.541	37 (62.7%)	41 (69.5%)	0.437
IGV1	38 (35.2%)	18 (30.5%)		22 (37.3%)	18 (30.5%)	
Mean size of fundal varix (mm)	25±9	25±6	0.922	26±9	25±6	0.771
No. of coils used	2 (1–8)	–	–	2 (1–8)	–	–
Glue/sclerosant injected (mL)	1 (0.5–3)*	4 (2–8) ^†^	–	1 (0.5–3)*	4 (2–8) ^†^	–
EHPVO, extrahepatic portal vein obstruction; EUS, endoscopic ultrasound; GOV2, gastroesophageal varices type 2; HBV, hepatitis B; HCV, hepatitis C; IGV1, isolated gastric varices type 1; MASLD, metabolic dysfunction-associated steatotic liver disease; NCPF, non-cirrhotic portal fibrosis; NCPH, non-cirrhotic portal hypertension; RTO, retrograde transvenous obliteration. *Glue. ^†^ Sclerosant.						

### Primary outcome

Table 2 compares primary and secondary outcomes between the two groups in the overall and matched cohort. Incidence of variceal bleeding within 1 year of the index procedure was comparable between the EUS and BRTO groups in the overall (13/108, 12.0% vs. 8/59, 13.6%; *P* = 0.777) and matched cohort (9/59, 15.3% vs. 13/59, 22.0%; *P* = 0.793). Median time to rebleeding was 16 weeks (1–40) in the EUS group and 8 weeks (1–50) in the RTO group ( *P* = 0.547). Similarly, incidence of bleeding from GVs was comparable between the EUS and BRTO groups in the overall (11/108, 10.2% vs. 2/59, 3.4%; *P* = 0.141) and matched cohort (8/59, 13.5% vs. 2/59, 3.4%; *P* = 0.094). Median time to rebleeding from GVs was 16 weeks (4–40) in the EUS group and 5 weeks (1–8) in the RTO group. Incidence of bleeding from esophageal varices was higher in the BRTO group (6/59, 10.2% vs. 2/108, 1.9%; *P* = 0.023), whereas it showed a trend toward significance in the matched cohort (6/59, 10.2% vs. 1/59, 1.7%; *P* = 0.061). Fig. 1 compares primary and secondary outcomes between the matched cohorts.

**Table:** **Table 2** Primary and secondary outcomes of procedures in the overall and matched cohort.

Parameters	Overall			Matched		
	EUS (n = 108)	RTO (n = 59)	*P* value	EUS (n = 59)	RTO (n = 59)	*P* value
Bleeding within 1 year	13 (12.0%)	8 (13.6%)	0.777	9 (15.3%)	8 (13.6%)	0.793
Gastric varices	11 (10.2%)	2 (3.4%)	0.141	8 (13.5%)	2 (3.4%)	0.094
Esophageal varices	2 (1.9%)	6 (10.2%)	**0.023**	1 (1.7%)	6 (10.2%)	0.061
Periprocedure adverse events	8 (7.4%)	13 (22.0%)	**0.006**	3 (5.1%)	13 (22.0%)	**0.007**
Bleeding	8 (7.4%)	0	0.051	3 (5.1%)	0	0.244
Worsening of ascites	0	9 (15.3%)	**0.000**	0	9 (15.3%)	**0.003**
Pulmonary embolism	0	1 (1.7%)	0.353	0	1 (1.7%)	1.000
Shunt rupture	0	2 (3.4%)	0.123	0	2 (3.4%)	0.496
Adrenal hemorrhage	0	1 (1.7%)	0.353	0	1 (1.7%)	1.000
GV obliteration at 4 weeks	87 (80.6%)	54 (91.5%)	0.062	49 (83.1%)	54 (91.5%)	0.167
Reintervention within 1 year	33 (30.6%)	13 (22.0%)	0.239	18 (30.5%)	13 (22.0%)	0.296
Gastric varices	31 (28.7%)	3 (5.1%)	**0.000**	17 (28.8%)	3 (5.1%)	**0.001**
Esophageal varices	2 (1.8%)	10 (16.9%)	**0.000**	1 (1.7%)	10 (16.9%)	**0.008**
Number of interventions for GV	1.30 ± 0.53	1.05 ± 0.22	**0.001**	1.25 ± 0.51	1.05 ± 0.22	**0.006**
Mortality within 1 year	10 (9.3%)	5 (8.5%)	0.865	6 (10.2%)	5 (8.5%)	0.751
EUS, endoscopic ultrasound; GV, gastric varix; RTO, retrograde transvenous obliteration.						

### Secondary outcomes

#### Procedure-related adverse events

Procedure-related AEs were seen in a higher proportion of patients in the RTO group, both in the overall (13/59, 22% vs. 8/108, 7.4%; *P* = 0.006) and matched cohort (13/59, 22% vs. 3/59, 5.1%; *P* = 0.007), although all the AEs were mild to moderate. Reported AEs in the BRTO group were new onset or worsening of ascites (9/59, 15.3%), shunt rupture (2/59, 3.4%), PE (1,59, 1.7%), and adrenal hemorrhage (1/59, 1.7%). Shunt rupture was insignificant in both cases, and the procedure was completed uneventfully. A case of PE was also clinically insignificant. One patient had intraprocedure adrenal hemorrhage due to a rupture of the adrenal vein, requiring adrenal artery embolization. Post-procedure bleeding on endoscopic visualization after EUS-guided intervention was reported in 7.4% cases (8/108). In three cases, bleeding stopped spontaneously, and five were managed with an additional endoscopic glue injection. Incidence of new onset or worsening of ascites was significantly higher in the RTO group, both in the overall (9/59, 15.3% vs. 0/108, 0%; *P* = 0.000) and matched cohort (9/59, 15.3% vs. 0/59, 0%; *P* = 0.003). However, all these episodes of ascites could be managed with diuretics.

#### Variceal obliteration at 4 weeks

Fundal variceal obliteration at 4 weeks was comparable between EUS and RTO in the overall (87/108, 80.6% vs. 54/59, 91.5%; *P* = 0.062) and matched cohort (49/59, 83.1% vs. 54/59, 91.5%; *P* = 0.167).

#### Reintervention within 1 year

**Fig. 2:**
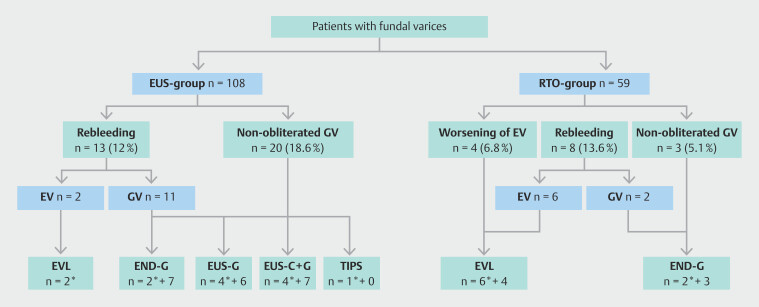
Flowchart showing details of type of reinterventions in both groups. END-G, endoscopic glue injection; EUS, endoscopic ultrasound; EUS-C+G, EUS-guided coil + glue injection; EUS-G, EUS-guided glue injection; EV, esophageal varices; EVL, endoscopic variceal ligation; GV, gastric varices; RTO, retrograde transvenous obliteration.

Need for reintervention within 1 year of the index procedure was comparable between EUS and RTO groups in the overall (33/108, 30.6% vs. 13/59, 22.0%; *P* = 0.239) and matched cohort (18/59, 30.5% vs. 13/59, 22.0%; *P* = 0.296). Fig. 2 shows the study flowchart with details of the type of reinterventions in both groups. However, need for reintervention for GVs was higher in the EUS group in the overall (31/108, 28.7% vs. 3/59, 5.1%; *P* = 0.000) and matched cohort (17/59, 28.8% vs. 3/59, 5.1%; *P* = 0.001). In the EUS group, reintervention for GVs was required in 25 of 91 (27.5%) in the EUS-C+G group and six of 17 (35.3%) in the EUS-G group ( *P* = 0.513). Similarly, need for reintervention for EVs was higher in the RTO group in the overall (10/59, 16.9% vs. 2/108, 1.8%; *P* = 0.000) and matched cohort (10/59, 16.9% vs. 1/59, 1.7%; *P* = 0.008).

#### Mortality within 1 year

There was no difference in mortality at 1 year between the EUS group and the RTO group in the overall (10/108, 9.3% vs. 5/59, 8.5%; *P* = 0.865) and matched cohort (6/59, 10.2% vs. 5/59, 8.5%; *P* = 0.751).

### Subgroup analysis

Subgroup analysis of matched cohorts based on procedure type and indication is summarized in Table 3 . Subgroup analysis was performed using matched data from patients undergoing EUS-C+G (n = 45) and PARTO (n = 45), which were the most common procedures in the respective groups. There was no difference between the groups in incidence of rebleeding, periprocedure AEs, GV obliteration at 4 weeks, reintervention at 1 year, or mortality within 1 year. However, incidence of new onset or worsening of ascites was significantly higher in the PARTO group (6/45, 13.3% vs. 0/59, 0%; *P* = 0.026). Need for reintervention for GVs was higher in the EUS group (11/45, 24.4% vs. 2/45, 4.4%; *P* = 0.014), whereas need for reintervention for EVs was higher in the PARTO group (9/45, 20.0% vs. 1/45, 2.2%; *P* = 0.015). When comparing data from patients undergoing EUS or RTO for a secondary indication, similar efficacy was observed with a higher incidence of worsening ascites in the RTO group and a higher incidence of reintervention for GV in the EUS group.

**Table:** **Table 3** Subgroup analysis of matched cohorts based on procedure type and indication.

Parameters	Procedure type			Secondary indication		
	EUS-C+G (n = 45)	PARTO (n = 45)	*P* value	EUS (n = 43)	RTO (n = 43)	*P* value
Rebleeding within 1 year	3 (6.7%)	7 (15.6%)	0.315	8 (18.6%)	6 (14.0%)	0.559
Gastric varices	2 (4.4%)	1 (2.2%)	1.000	4 (9.3%)	1 (2.3%)	0.360
Esophageal varices	1 (2.2%)	6 (13.3%)	0.110	4 (9.3%)	5 (11.6%)	1.000
Periprocedural adverse events	4 (8.9%)	10 (22.2%)	0.144	5 (11.6%)	8 (18.6%)	0.366
Bleeding	4 (8.9%)	0	0.116	5 (11.6%)	0	0.055
Worsening of ascites	0	6 (13.3%)	**0.026**	0	6	**0.025**
Pulmonary embolism	0	1 (2.2%)	1.000	0	0	1.000
Shunt rupture	0	2 (4.4%)	0.494	0	1 (2.3%)	1.000
Adrenal hemorrhage	0	1 (2.2%)	1.000	0	1 (2.3%)	1.000
GV obliteration at 4 weeks	38 (84.4%)	42 (93.3%)	0.180	39 (90.7%)	40 (93.0%)	1.000
Reintervention within 1 year	12 (26.7%)	11 (24.4%)	0.809	13 (30.2%)	8 (18.6%)	0.209
Gastric varices	11 (24.4%)	2 (4.4%)	**0.014**	9 (20.9%)	1 (2.3%)	**0.014**
Esophageal varices	1 (2.2%)	9 (20.0%)	**0.015**	4 (9.3%)	7 (16.3%)	0.052
Mortality within 1 year	6 (13.3%)	2 (4.4%)	0.266	4 (9.3%)	4 (9.3%)	1.000
EUS, endoscopic ultrasound; EUS C+G, EUS-guided coil + glue; GV, gastric varix; PARTO, plug-assisted retrograde transvenous obliteration; RTO, retrograde transvenous obliteration.						

### Predictors of rebleeding and reintervention within 1 year

Analysis of factors associated with outcome of bleeding or reintervention within 1 year was done using a multivariable model ( Table 4 ). Parameters included were presence of cirrhosis, prior bleeding, GOV2, RTO, and size of the varix. Among these parameters, prior bleeding was found to be the only independent predictor of bleeding within 1 year (OR 3.666, 95%CI 1.013–13.272, *P* = 0.048). Similarly, EUS-guided intervention was an independent predictor of reintervention within 1 year (OR 3.140, 95% CI 1.309–7.534, *P* = 0.010).

**Table:** **Table 4** Multivariable analysis of factors associated with bleeding or reintervention within 1 year after the index procedure.

Parameters	Rebleeding		Reintervention	
	Odds ratio (95% CI)	*P* value	Odds ratio (95% CI)	*P* value
Presence of cirrhosis	1.184 (0.330–4.253)	0.795	1.072 (0.412–2.790)	0.887
Prior bleeding	3.666 (1.013–13.272)	0.048	1.446 (0.615–3.399)	0.398
GOV2	0.849 (0.297–2.426)	0.760	1.044 (0.984–1.079)	0.201
EUS-guided intervention	1.020 (0.384–2.711)	0.968	3.140 (1.309–7.534)	**0.010**
Varix size	1.014 (0.958–1.073)	0.635	1.024 (0.980–1.070)	0.282
CI, confidence interval; EUS, endoscopic ultrasound; GOV2, gastroesophageal varices type 2.				

## Discussion

Both RTO and EUS-C+G have been demonstrated to have lower rates of rebleeding and reintervention in fundal varices compared with endoscopic glue injection 8 12 13 14 . However, studies comparing RTO with EUS-guided intervention for fundal varices are limited. The present study reported a comparable incidence of variceal obliteration at 4 weeks, as well as variceal bleeding and bleeding from GVs within 1 year of the index procedure between the EUS and RTO groups in the overall and matched cohort. Although incidence of bleeding from EVs was higher in the RTO group, it was comparable in the matched cohort. Procedure-related AEs, primarily new onset or worsening of ascites, were seen in a higher proportion of patients in the RTO group, both in the overall and matched cohort. Need for reintervention within 1 year of the index procedure was comparable between the EUS and RTO groups in the overall and matched cohort. However, need for reintervention for GVs was higher in the EUS group, and need for reintervention for EVs was higher in the RTO group.

A previous meta-analysis pooling data on EUS-guided therapies reported a GV obliteration rate of 84.4% (95% CI 74.8–90.9). Pooled rates of recurrence of GVs, early rebleeding, and late rebleeding were 9.1% (95% CI 5.2–5.7), 7.0% (95% CI 4.6–10.7), and 11.6% (95 % CI 8.8–15.1) 15 . After BRTO, reported incidence of bleeding from GVs was 10% to 20%, whereas bleeding from all varices was 19% to 31% 16 . Thus, reported incidence of rebleeding remains comparable after either of the procedures. Overall rebleeding rates (EUS 15.3% vs. BRTO 13.6%; *P* = 0.793) observed in this study are consistent with findings from a previously published retrospective study comparing EUS-C+G and BRTO for GVs with spontaneous portosystemic shunt, which reported 1-year all-cause bleeding rates of 20% and 18.9% ( *P* = 0.9), respectively 17 . Multivariate analysis in our study revealed that prior bleeding from GV was a significant predictor of bleeding within 1 year, which highlights the need for special attention and tailored therapeutic approaches for high-risk populations.

The present analysis showed that both EUS-guided intervention and RTO offer comparable efficacy for managing fundal varices regarding variceal obliteration and preventing bleeding. Huang et al. reported a similar 1-year all-cause rebleeding rate (20.0% vs. 18.9%, *P* = 0.900) and 1-year mortality rate (2.0% vs. 0%, *P* = 1.000) with EUS-C+G and BRTO 17 . In a previous network meta-analysis, there was no significant difference between EUS-guided intervention and BRTO in the indirect comparison with respect to variceal obliteration, bleeding, moderate-severe AEs, or mortality 8 . These results reinforce the utility of EUS-guided therapies as an effective alternative to RTO, particularly in centers with access to skilled endosonographers.

The proportion of patients requiring reintervention was comparable between the EUS and RTO groups in the overall and matched cohort. However, need for reintervention for GVs was higher in the EUS group. This may be due to the fact that in RTO, portions of the afferent and efferent veins are obliterated along with the fundal varices 18 . In contrast, EUS-guided intervention involves mostly injection into the varix, leaving intact afferents and efferents, which may lead to redevelopment of varices. Lastly, there may be incomplete obliteration of the fundal varices, leading to recurrence.

Aggravation of esophageal varices appears to be a major problem following RTO and reflects post-procedure increased portal blood flow and pressure. The reported rate of aggravation of esophageal varices after BRTO ranges from 14% to 68% 16 . Thus, patients undergoing BRTO should be placed on scheduled endoscopic surveillance for EVs. BRTO has been associated with worsening of ascites in 0% to 43.5% of cases 16 . PARTO is being increasingly used in place of BRTO due to shorter procedure duration and lower incidence of AEs 5 19 . A study on the outcome of PARTO reported worsening of ascites in 16.7% of cases and worsening of EV status in 53.1% of cases 20 . In the present study, reported AEs in the BRTO group were new onset or worsening of ascites (15.3%), shunt rupture (3.4%), pulmonary embolism (PE) (1.7%), and adrenal hemorrhage (1.7%). In comparison, the EUS group was associated with only post-procedure bleeding (7.4%). This post-procedure bleeding may be due to incomplete obliteration of varices, and thus, sufficient care should be taken to document complete absence of Doppler flow to reduce risk of post-procedure bleeding. Of note, all but one of the non-ascites complications were clinically insignificant. Incidence of new onset or worsening of ascites was significantly higher in the BRTO group, both in the overall (15.3% vs. 0%; *P* = 0.000) and matched cohort (15.3% vs. 0%; *P* = 0.003). All cases of ascites following RTO responded well to diuretic therapy. Thus, BRTO is associated with a higher risk of AEs than EUS-guided intervention, although none of these were severe.

Selection of techniques for management of fundal varices is influenced by multiple factors, the most important being anatomy of fundal varices, as assessed by CT. RTO may not be feasible in Kiyosue type D varices (multiple efferent collateral veins without large shunts), and EUS-guided therapy may be beneficial in such cases 18 . Shunt size and angle may predict RTO technical success. The maximal available plug diameter is 22 mm, and thus, shunts > 18 mm at the left renal vein confluence are not routinely occluded by PARTO 21 . Conversely, small shunts and shunts with acute angulation can limit access to intervention. EUS-guided therapy and CARTO may be useful in both smaller and larger vessels that are not amenable to plug or balloon placement 21 . In a previous study, hypoalbuminemia and Child-Pugh B/C status were independent predictors of ascites after BRTO 22 . In another study, liver stiffness measurement ≥ 21 kPa was a significant independent risk factor for worsening of hepatic venous pressure gradient and poor prognosis after BRTO 23 . In patients with active bleeding with blood in the fundus precluding endoscopic visualization, EUS-guided intervention may offer emergency management of fundal varices. Lastly, the number of interventional gastroenterologists is significantly higher compared with interventional radiologists in India, limiting access to RTO 24 25 . Thus, anatomy of varices, indication (emergency vs. elective), patient status (e.g., presence of ascites, Child-Pugh status), and available expertise should be considered in decision-making about therapy for managing fundal varices.

The present study is one of the few to compare outcomes of EUS-guided therapies versus standard and modified BRTO for management of fundal varices. Use of propensity score matching adds to data robustness. Despite these strengths, there are a few limitations. First, a retrospective study design increases risk of selection bias. Second, EUS-guided interventions in the study included both EUS-G and EUS-C+G. EUS-C+G reportedly is associated with a lower rebleeding risk compared with EUS-G, as documented in a previous meta-analysis 8 . Similarly, PARTO has been shown to have a higher recurrence rate than BRTO in a previous study. Thus, significant heterogeneity with respect to treatment modality may be a limitation. Subgroup analysis partially addresses this limitation but emphasizes the need for randomized trials. Third, involvement of multiple operators with different expertise may add to heterogeneity. Fourth, a cost-effectiveness analysis with regard to the procedure, reintervention, and hospitalization due to AE or rebleeding could not be performed due to significant variations in cost of the procedures and consumables across the centers. Lastly, a longer follow-up duration would have helped better analyze long-term outcomes of both procedures.

## Conclusions

To conclude, EUS-guided therapies for fundal varices are a safer alternative compared with RTO, with a significantly lower incidence of AEs and comparable efficacy. However, their higher reintervention rate for GVs demands standardization of the technique and vigilant follow-up. Although BRTO is effective, it carries a higher risk of worsening ascites and EV aggravation. Prior bleeding from GV predicted bleeding within 1 year, whereas EUS-guided intervention was an independent predictor of reintervention within 1 year. These factors should be considered while determining the optimal therapy for patients with fundal varices. Further RCTs with longer follow-up duration are needed to validate findings from the present study.
